# 

**Published:** 1879-01

**Authors:** 


### Editorial.

### ALUMNI RE-UNION.

In the following it will be seen that the preliminary steps have been taken for a re-union of the alumni of the Ohio College of Dental Surgery, to be held in March next at the close of the college session.

It is very desirable that every class should be as fully represented as possible, that the object of the meeting may be realized. And especially is it important that the member of each class appointed upon the general committee attend well to the duty assigned him. Each one should use his best effort to have his class fully represented, and let the report from each class be accurate and complete.

If any one appointed upon the general committee finds it impossible for him to perform the duty, he should at once notify the executive committee, whose names are given in the circular, that another may be appointed.

"At the close of the last session of the Ohio College of Dental Surgery, it was decided by those interested who were present, to hold a re-union of all the Alumni at the time of the next annual commencement when it is proposed to have every class that has ever gone out from the Institution, as fully represented as possible. The object of this re-union, is to bring together the graduates of the college upon the Tercentenary Anniversary, that they may revive acquaintance and pledge themselves anew to give her that support and aid, which enhances the usefulness of educational institutions.

A general committee consisting of one member from each class, was appointed, whose duty it shall be to interest their respective classes in the meeting, and to furnish biographical sketches of deceased members. They will also give the leading items of interest pertaining to the classes, present location, status, etc., of the members, the reports to be made in the order of graduation.

The executive committee appointed, will attend to all arrangements necessary for the success of the meeting and entertainment of those attending. It is earnestly desired that every one will respond to this call, and by his presence give assurance of continued interest in his time-honored Alma Mater.

General Committee.—A, Berry, of Cincinnati, O., of class 1846 ; A. S. Talbert, Lexington, Ky., 1847 ; J. S. King, Pittsburg, Pa., 1848; E. Bray, Evansville, Ind., 1849; J. Ramsey, Springfield, O., 1850; J. C. Clark, Circleville, O., 1851; J. C. Whinnery, Salem, O., 1852; Joseph Richardson, Terre Haute, Ind., 1853; E. Collins, Little Rock, Ark., 1854; R. C Reid, Anderson, Ind., 1855; E. Osmond, Cincinnati, O., 1856; H. A. Smith. Cincinnati, O., 1857; S. L. Bracy, Raymond

Miss., 1858, E. Ruth, Maysville, Ky., 1859; J. A. Watling, Ypsilanti, Mich., 1860; A, Evans. Hillsboro, O., 1861; Wm. Taft, Cincinnati, O., 1863; W. N. Morrison, St. Louis, Mo., 1864; J. I. Taylor, Cincinnati, O., 1865; C. C. Chittenden, Madison, Wis., 1866; Henri L. Ambler, Cleveland, O., 1867; J. P. Holmes, Terry, Miss., 1868; J. S. Cassidy, Covington, Ky., 1869; J. M. Porter, Toledo, O., 1870; J. E. Cravens, Indianapolis, 1871; J. H. Warner, Columbus, O., 1872; T. S. Hacker, Indianapolis, Ind., 1863; L. L. Dunbar, San Francisco, Cal., 1864; C. B. Mower, Wooster, O., 1875; C, I. Keely, Oxford, O., 1876; A. L. Brown, Cincinnati, O., 1877; Thos. A. Stephenson, Cincinnati, O., 1868.

Executive Committee.—J. Taft, Chairman, J. S. Cassidy, H. A. Smith, E. G. Betty, Secretary.

Programme—Thursday, March, 6, 1879, 2. p. m. Organization of meeting. Calling the roll. Miscellaneous business. Historical Address, Dr. Geo. Watt, class 1854. Reading of reports from General committees in the order of graduation. The evening to be devoted to an entertainmaint, sentiments, toasts, etc."

Effort is being made by the Faculty to extend and render attractive the Museum of the College. The co-operation of the Alumni is earnestly desired in this work.

Donations of specimens and models of anomalous cases are respectfully solicited. Send to H. A. Smith, Dean, 286 Race St., when suitable acknowledgment and thanks for the same will be made.

*THE DENTAL REGISTER,*

A MONTHLY MAGAZINE DEVOTED TO THE INTERESTS OF

THE DENTAL PROFESSION.

*Terms Reduced to $2 50 per Year. Single Copies 25c.*

*EDITED BY PROF. J. TAFT, D. D. S.,*

AND PUBLISHED BY

SPENCER & CROCKER, Ohio Dental Depot.

The volume commences in January and closes in December.

The REGISTER being issued monthly is always fresh, therefore Indispensable to the practitioner who desires to keep posted up with the new inventions and improvements which are daily developed. Neither expense nor effort will be spared to give value and variety to its contents. Articles will be illustrated with well executed engravings whenever they will add to the interest or assist to explanation.

Its extensive circulation and frequency of issue make it one of the BEST DENTAL ADVERTISING MEDIUMS in the United States.

All subscriptions must begin with January or July.

Local or special notices, five lines of less, will be inserted for $3.00 each insertion; over five lines, 50 cts. per line.

All advertising bills payable in advance, or at furthest, after the issue of the first number containing the advertisement. All transient advertisements to be paid for in advance.

☞ CONTRIBUTIONS SOLICITED.

Exclusively Editorial Communications should be addressed to Editor.

The latest number of the REGISTER may be ordered with or without other goods at any time, and all letters should be addressed to

**SPENCER & CROCKER, Ohio Dental Depot, Cincinnati, O.**

